# Design and Optimization of a Chemometric-Assisted Spectrophotometric Determination of Telmisartan and Hydrochlorothiazide in Pharmaceutical Dosage Form

**DOI:** 10.4103/0975-1483.62224

**Published:** 2010

**Authors:** KS Lakshmi, S Lakshmi

**Affiliations:** *Department of Pharmaceutical Analysis, SRM College of Pharmacy, SRM University, Kattankulathur - 603 203, Tamil Nadu, India*

**Keywords:** Chemometrics, hydrochlorothiazide, partial least square, principal component regression, telmisartan

## Abstract

Two chemometric methods were developed for the simultaneous determination of telmisartan and hydrochlorothiazide. The chemometric methods applied were principal component regression (PCR) and partial least square (PLS-1). These approaches were successfully applied to quantify the two drugs in the mixture using the information included in the UV absorption spectra of appropriate solutions in the range of 200-350 nm with the intervals Δλ = 1 nm. The calibration of PCR and PLS-1 models was evaluated by internal validation (prediction of compounds in its own designed training set of calibration) and by external validation over laboratory prepared mixtures and pharmaceutical preparations. The PCR and PLS-1 methods require neither any separation step, nor any prior graphical treatment of the overlapping spectra of the two drugs in a mixture. The results of PCR and PLS-1 methods were compared with each other and a good agreement was found.

## INTRODUCTION

Telmisartan (TEL) is angiotensin-II receptor antagonist used in the treatment of hypertension.[1] Hydrochlorothiazide (HCZ) is one of the oldest and widely used thiazide diuretic.[2] Many analytical methods were developed for its determination either alone[3,4] or in combination with other antihypertensive drugs. These methods include spectrophotometry,[5–7] second derivative and first derivative of ratio spectra[8] absorbance ratio and first derivative,[9] polarography,[10] flow injection analysis,[11] HPLC,[12–14] and HPTLC.[15] The literature revealed some methods which include spectrophotometry[16] for the simultaneous determination of both the drugs.

Under computer controlled instrumentation, multivariate calibration methods are playing a very important role in the multicomponent analysis of mixtures by UV-VIS molecular absorption spectrophotometry. The approach is useful in the resolution of band overlapping in quantitative analysis. The multivariate calibration has been found to be the method of choice for complexed mixtures. The advantage of multicomponent analysis using multivariate calibration is the speed of the determination of the components in a mixture, avoiding a preliminary separation step.[17] Control analysis on pharmaceutical preparations using the multivariate calibration method have been proved to be a valid alternative to HPLC.[18]

The aim of this paper is to investigate the ability of PLS-1 and PCR methods to quantify a binary mixture of TEL and HCZ with overlapping UV spectra and to apply the optimized models[19] in pharmaceutical preparations. The proposed methods are simple and accurate. They resulted in a significant reduction in analysis time and proved to be suitable for routine determination of the two components of the standard mixture.

## EXPERIMENTAL DETAILS

### Reagents and materials

Pharmaceutical grades TEL and HCZ were obtained from Madras Pharmaceuticals Pvt, Ltd, Chennai, as gift sample. Pharmaceutical preparation containing TEL and HCZ (Telista-H containing 40 mg TEL and 12.5 mg HCZ/tablet) were obtained from local pharmacies. All other chemicals were analytical reagent grade.

### Instrumentation

A Perkin Elmer (Lamda 25) spectrophotometer controlled by a computer and equipped with a 1 cm pathlength quartz cell was used for UV-Vis spectra acquisition. Spectra were acquired between 200 and 350 nm (2 nm resolution). PLS-1 and PCR analyses were carried out by using PLS-Toolbox software version 5.0-PC for use with Matlab 7.5.

### Standard solutions and calibration

Standard solutions of each TEL and HCZ were prepared separately dissolving 100 mg of each drug in 100 ml of 0.1 M sodium hydroxide and then further dilutions were made with water within the concentration range of 1-6 μg mL^–1^for TEL and 0.5-2.5 μg mL^–1^for HCZ [Table 1]. The UV absorption spectra were recorded over the wavelength range of 200-350 nm. The data points of the spectra were collected every 1 nm. The computation was made using PLS-Toolbox software version 5.0. The PLS-1 and PCR models were applied to the UV absorption spectra of these mixtures using nine latent variables for TEL and HCZ by PLS-1. Nine principal components were used for PCR determination of each component.

**Table 1:** Concentration data for the different mixtures used in the calibration set and internal validation for the determination of telmisartan and hydrochlorothiazide using partial least square and principal component regression methods

Mixture No.	Mixture composition (μg/mL)		Internal validation (% Recovery)			
	TEL	HCZ	PLS-1		PCR	
			TEL	HCZ	TEL	HCZ
1	1.0	0.5	103.44	99.78	103.21	98.64
2	1.0	1.0	101.93	93.02	100.25	92.43
3	1.0	1.5	101.14	99.77	101.29	96.44
4	1.0	2.0	101.40	101.70	99.85	103.84
5	2.0	0.5	99.87	95.12	98.95	95.26
6	2.0	1.0	95.55	104.33	95.78	103.39
7	2.0	1.5	106.55	97.08	107.15	96.28
8	2.0	2.0	99.93	101.77	100.32	101.01
9	3.0	2.5	99.65	100.54	99.85	104.15
10	3.0	2.0	99.25	97.98	100.15	97.57
11	3.0	1.5	99.07	99.37	99.26	99.02
12	3.0	1.0	98.62	106.79	98.68	86.58
13	4.0	0.5	102.17	102.26	102.53	101.98
14	4.0	1.0	100.16	102.84	100.01	101.87
15	4.0	1.5	99.82	101.18	100.02	100.38
16	4.0	2.0	100.67	99.43	101.04	94.62
17	5.0	2.5	99.45	98.91	98.82	98.11
18	5.0	1.5	99.62	102.28	99.43	100.92
19	5.0	0.5	97.70	99.18	97.56	99.94
20	5.0	1.0	100.63	99.39	100.56	106.51
21	1.5	1.0	99.58	109.38	93.02	103.21
22	2.5	2.0	100.97	95.84	100.95	97.47
23	3.5	1.0	99.99	103.25	100.35	107.12
24	4.5	1.0	101.86	95.09	101.84	98.74
25	3.5	2.0	100.05	100.48	99.74	103.81
Mean^a^			100.36	100.27	100.02	99.57
S.D^a^			2.017	3.637	2.526	4.573

a Mean and standard deviation (S.D); Percentage recovery with respect to the actual concentration

### Sample preparation

Twenty tablets were weighed and finely powdered. An accurately weighed portion of the powder equivalent to about 40 mg of TEL and 12.5 mg of HCZ was extracted and diluted to 100 ml with 0.1 M sodium hydroxide. The sample solution was filtered. Further dilution of the filtrate was carried out with water to provide a solution of 4 μg mL of TEL and 1.25 μg/mL of HCZ.

### Procedures for the determination of telmisartan and hydrochlorothiazide using partial least square and principal component regression methods

The UV absorption spectrum of final solution was recorded over the wavelength range of 200-350 nm. The data points of the spectrum were collected every 1 nm. The PLS-1 model was applied using nine latent variables for TEL and HCZ. The PCR model was applied using nine principal components. The concentration of each component was calculated using each model.

## RESULTS AND DISCUSSION

**Figure 1:**
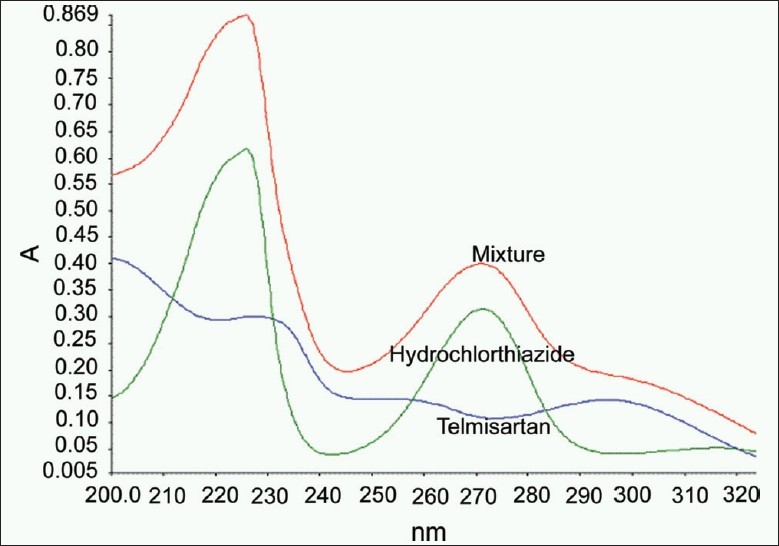
UV absorption spectra of telmisartan, hydrochlorothiazide, and the mixture

Figure 1 shows the UV absorption spectra of TEL and HCZ at their nominal concentrations in the tablet. A significant overlap in absorption bands was noticed. The simultaneous determination of TEL and HCZ in the tablet by conventional, derivative, and derivative ratio spectrophotometric methods is hindered by strong spectral overlap throughout the wavelength range. The PLS or PCR calibration methods were necessary for such determination due to the presence of interference.

A training set was designed in 25 laboratory made sample mixtures in the concentration range of 1-5 μg/mL^1^for TEL and 0.5-2.5 μg/mL for HCZ in PCR and PLS-1 methods [Table 1]. The absorbance data matrix were obtained by measuring the absorbances between 200 and 350 nm in the intervals as Δλ = 1 nm at 151 wavelengths in PCR and PLS-1 in the zero-order absorption spectra. The model was built with the help of the software. The concentrations of the components present in the different sample mixtures were then estimated with the help of the model. The predicted concentrations of the components in each sample were then compared with the actual concentrations in these training samples and the root mean square error of cross-validation (RMSEC) was calculated for each method as follows:

$$\mathrm{RMSEC}\;=\;{\left(\mathrm{PRESS}/n\right)}^{1/2}$$

where *n* is the number of training samples.

$$\mathrm{PRESS}\;=\;\Sigma \;{\left(Y_{\mathrm{pred}}-Y_{\mathrm{true}}\right)}^{2}$$

where Y_pred_ and Y_true_ are predicted and true concentration in μg/mL, respectively.

The RMSEC was used as a diagnostic test for examining the errors in the predicted concentrations. It indicates both the precision and accuracy of predictions. The RMSEC plays the same role of concentration errors. The selected model is the one with the smallest number of factors such that RMSEC for that model is not greater than RMSEC for the model with additional factor. Satisfactory results were obtained for each compound in the training set by PLS-1 and PCR optimized models indicating good predictive abilities of the models. The obtained results are shown in [Table 2] indicating good accuracy and precision.

**Table 2:** Root mean square error of cross-validation and statistical parameter values for simultaneous determination of telmisartan and hydrochlorothiazide using partial least square and principal component regression methods

Parameter	Method	Compound	
		TEL	HCZ
RMSEC	PLS-1	0.0539	0.0417
	PCR	0.0572	0.1068
Correlation coefficient	PLS-1	0.9987	0.9956
	PCR	0.9982	0.9910
Slope	PLS-1	0.9951	0.9904
	PCR	0.9982	1.0054
Intercept	PLS-1	0.0188	0.0152
	PCR	0.0054	0.0115

### Application in synthetic and real samples

The proposed methods were applied to the simultaneous determination of TEL and HCZ in commercial tablets (Telista-H). Tables 3 and 4 show the results obtained by the application of the PCR and PLS models on the prediction sets and a pharmaceutical formulation (Telista-H tablet). Five replicate determinations were carried out on each experiment. These results confirm satisfactory to the label claim, synthesized concentration, and indicate the high precision and accuracy of the proposed methods when applied to tablets.

**Table 3:** Composition of prediction set, their predictions by partial least square and principal component regression models

Sample no.	Composition (μg/mL)		% Recovery			
	TEL	HCZ	PLS-1		PCR	
			TEL	HCZ	TEL	HCZ
1	1.0	2.0	96.60	97.72	95.87	97.37
2	2.0	2.5	94.79	99.67	94.86	98.96
3	4.0	2.0	103.56	103.81	103.14	106.67
4	4.0	2.0	103.56	103.81	103.14	106.67
5	5.0	2.0	105.44	89.85	104.81	98.38
6	1.5	2.0	101.16	89.68	105.61	93.12
7	2.5	2.5	95.27	96.06	91.79	93.85
8	3.5	1.5	104.91	97.58	104.74	107.77
9	3.0	2.5	99.65	100.54	99.85	104.15
10	1.5	1.5	97.68	97.68	97.55	95.49
Mean^a^			100.70	97.49	100.57	99.95
S.D^a^			4.635	4.704	5.453	5.297

a Mean and standard deviation; Percentage recovery with respect to the composition

**Table 4:** Determination of telmisartan and hydrochlorothiazide in commercial tablets using partial least square and principal component regression methods

Sample no.	Composition (μg/mL)		%Recovery			
	TEL	HCZ	PLS-1		PCR	
			TEL	HCZ	TEL	HCZ
2	2.0	0.62	95.31	97.25	95.25	98.96
3	3.0	0.93	106.15	101.25	107.92	106.42
4	4.0	1.25	103.56	89.99	101.74	103.81
5	5.0	1.56	98.58	103.44	102.58	98.36
Mean^a^			100.14	98.32	102.32	101.06
S.D^a^			4.546	5.175	4.611	3.813

a Mean and standard deviation (S.D); Percentage recovery with respect to the label claim

### Accuracy

This study was performed by adding known amounts of the studied compounds to a known concentration of the commercial pharmaceutical tablets (standard addition method). The resulting mixtures were analyzed and results obtained were compared with the expected results. The excellent recoveries of the standard addition method [Table 5] suggested the high accuracy of the proposed methods.

**Table 5:** Application of standard addition technique to the analysis of telmisartan and hydrochlorothiazide using partial least square and principal component regression methods

Sample no.	Composition (μg/mL) TEL		% Recovery	
	Claimed	Added	PLS-1	PCR
1	1.0	0.5	99.23	103.44
2	1.0	1.0	97.42	101.62
3	1.0	1.5	98.35	101.25
4	1.0	2.0	103.56	89.99
5	1.0	2.5	104.22	103.48
Mean^a^			100.55	99.95
S.D^a^			3.118	5.663
Composition (μg/mL) HCZ				
1	0.5	0.5		
2	0.5	1.0	97.68	89.96
3	0.5	1.5	105.12	101.25
4	0.5	2.0	101.88	92.74
5	0.5	2.5	104.32	101.68
Mean^a^			102.04	97.12
S.D^a^			2.931	5.396

a Mean and standard deviation (S.D); Percentage recovery from the added amount

### Precision

The precision was determined by means of a one-way ANOVA including 10 replicates carried out on three successive days using two chemometric methods for synthetic mixtures. Snedecor F values below the tabulated levels were obtained in all cases (F = 4.15, n_1_= 2, n_2_= 27, Table 6) so there were no significant differences between the result obtained in the determination of each drug in the presence of the other on different days. The highest RSD (%) values were obtained for the PCR method for the between days and within days results for both TEL and HCZ.

**Table 6:** Analysis of variance (ANOVA) for the proposed methods

Parameters	PLS-1		PCR	
	TEL	HCZ	TEL	HCZ
Between days variance	5.68	6.33	5.88	8.78
Within days variance	3.55	4.57	7.78	9.24
F-ratio	1.60	1.38	1.34	1.05
Mean value	3.99	2.05	4.06	2.07
Between days RSD (%)	1.62	2.12	2.65	3.23
Within days RSD (%)	1.21	1.57	2.88	4.24

Between-day and within-day degrees of freedom 2 and 27, respectively. The critical *F*-ratio value for 2 and 27 degrees of freedom at 95% confidence level is 4.21

## CONCLUSION

The proposed methods based on processing the spectral data could be applied to the simultaneous determination of TEL and HCZ in mixtures and the pharmaceutical formulation selected containing its binary mixture without interference of each other. Chemometric methods are less expensive by comparison and they do not require sophisticated instrumentation and any prior separation step. But they need software for resolution and determination of the components of the mixture. The chemometric methods proposed are very powerful techniques for the simultaneous analysis of multicomponent mixtures in which the spectra of the active compounds overlap with each other and also, by the fact that zero-order spectra is enough for the analysis, there is no need for the spectrophotometer to have any other modes such as derivation and ratio spectra. The proposed methods, PLS-1 and PCR, were found to be suitable for the routine analysis of the component of pharmaceutical preparations containing TEL and HCZ.
